# Antiviral effects of *Codonopsis pilosula* extract and its bioactive components against porcine epidemic diarrhea virus in vitro via the AMPK/mTOR pathway

**DOI:** 10.1186/s12917-026-05477-w

**Published:** 2026-04-24

**Authors:** Tao Ren, Shiqin Pan, Xuqin Song, Liting Cao, Anchun Cheng, Yujie Zhan, Jian Yang, Deyuan Ou

**Affiliations:** 1 Institute of Veterinary Medicine and Immunology Drugs, Veterinary Department in College of Animal Science, State Key Laboratory of Green Pesticide, Guizhou University, Guiyang, 550025 China; 2 Qixingguan District Grassland Workstation, Bijie, 551700 China; 3 Department of Traditional Chinese Veterinary Medicine, College of Veterinary Medicine, Southwest University, Rongchang, Chongqing, 402460 China

**Keywords:** *Codonopsis pilosula*, IPEC-J2 cells, Porcine epidemic diarrhea virus, AMPK/mTOR pathway, Autophagy

## Abstract

**Background:**

Porcine Epidemic Diarrhea Virus (PEDV) frequently outbreaks across China, posing a significant economic threat to the swine industry. PEDV exhibits a high degree of variability and is associated with a high mortality rate in piglets. Currently, vaccines show limited efficacy against variant strains, making it critical to explore alternative treatments, particularly the potential of traditional Chinese medicine (TCM) in combating PEDV infection.

**Results:**

This study aims to elucidate the antiviral mechanisms of *Codonopsis pilosula* (Dangshen) aqueous extract and its bioactive components, with a particular focus on the relationship between autophagy and antiviral effects, thereby providing theoretical support for the clinical application of TCM in treating PEDV infection. In this study, different concentrations of *Codonopsis* aqueous extract (1.25 mg/mL, 2.5 mg/mL, and 5 mg/mL) were applied to PEDV-infected IPEC-J2 cells. The results demonstrated that cells treated with the high-dose group exhibited well-preserved cellular structure, with minimal organelle damage. Furthermore, the high-dose treatment significantly reduced the expression of PEDV N, LC3, AMPK, and p-AMPK proteins in the infected cells. Additionally, it modulated the expression of key regulators in the AMPK/mTOR pathway, including mTOR, while upregulating the expression of *AMPK* and *ATG13* mRNA. Moreover, five bioactive components of *Codonopsis* including β-sitosterol (SITO), quercetin (Que), 5-hydroxymethylfurfural (HMF), apigenin (APG), and nicotinic acid (NA), significantly decreased both the mRNA and protein expression of PEDV N and LC3. APG exhibits the strongest combined antiviral and anti-autophagic effects. SITO, QUE, APG, and NA reduced AMPK expression. QUE increased ATG13 levels, whereas the other four bioactive components significantly decreased ATG13 expression.

**Conclusions:**

Therefore, *Codonopsis* aqueous extract and its bioactive components can alleviate pathological damage in IPEC-J2 cells, inhibit PEDV replication, and modulate the expression of genes and proteins associated with the AMPK/mTOR pathway. This study provides new theoretical evidence for the potential clinical application of *Codonopsis* in the treatment of PEDV infection.

**Supplementary Information:**

The online version contains supplementary material available at 10.1186/s12917-026-05477-w.

## Introduction

Porcine epidemic diarrhea virus (PEDV) remains a major threat to the pig industry and continues to hinder the sustainable development of swine production in China [1]. PEDV is particularly devastating in suckling piglets, in which it causes high morbidity and mortality [2]. The prevalence of PEDV is higher than that of several other major porcine enteric pathogens, such as transmissible gastroenteritis virus (TGEV) and porcine rotavirus (PoRV) [3]. In addition, some PEDV variant strains harbor multiple insertions, deletions, and substitutions in structural proteins, which may alter viral antigenicity. As a result, conventional vaccines often provide insufficient protection against emerging variants, thereby contributing to recurrent outbreaks [4]. Although several experimental agents have shown anti-PEDV activity, broadly effective antiviral options for PEDV remain limited.

Autophagy is an evolutionarily conserved intracellular degradation process that participates in multiple stages of virus–host interaction, including viral entry, replication, and release [5]. Many viruses exploit autophagy to facilitate intracellular survival and propagation, thereby enhancing viral replication and pathogenicity [6]. LC3, a key autophagy-related protein, is essential for autophagosome formation and is frequently involved in the regulation of virus-induced autophagy [7]. In PEDV infection, viral proteins such as nsp6 and ORF3 have been reported to induce autophagy in IPEC-J2 cells, and PEDV-associated autophagy appears to promote viral replication [8]. In addition, PEDV infection can activate AMPK- and ULK1-related signaling, further supporting the involvement of autophagy in viral growth [9]. Because the AMPK/mTOR axis is a central regulator of autophagy, this pathway may be closely related to PEDV pathogenesis and may represent a potential target for antiviral intervention [10–12].

*Codonopsis pilosula* (Dangshen, DS), a widely used traditional Chinese medicinal herb, has attracted increasing attention because of its broad pharmacological activities [13, 14]. Previous studies have shown that DS and its bioactive constituents possess multiple biological effects, including immunomodulatory, gastrointestinal motility-regulating and gastroprotective, hepatoprotective, antioxidant, anti-inflammatory, hypoglycemic, hypolipidemic, antitumor, and antiviral activities [14, 15]. DS is considered relatively nontoxic and has few reported side effects [16]. Notably, DS saponins have been reported to alleviate intestinal inflammation, whereas DS flavonoids may promote the repair of gastrointestinal mucosal injury [17, 18]. In addition, DS has been implicated in the regulation of cardiovascular, digestive, and immune-related diseases through mechanisms involving inflammatory responses, oxidative stress, immune modulation, and apoptosis [15]. These properties suggest that DS may be of particular interest in enteric diseases characterized by epithelial injury, inflammation, and impaired mucosal barrier function.

Several bioactive constituents of DS may also be relevant to anti-PEDV research. DS contains flavonoids, organic acids, and other compounds, including quercetin and nicotinic acid [19]. Previous studies have shown that some flavonoids, including quercetin and apigenin [20], can inhibit PEDV replication, and that quercetin 7-rhamnoside suppresses PEDV infection during the early stage of viral replication [21–23]. Nicotinic acid has also been reported to reduce PEDV replication and intestinal injury in infected piglets by modulating host immune responses [24]. In addition, currently reported anti-PEDV strategies such as tomatidine, surfactin-derived lipopeptides, and probiotic-derived extracellular vesicles indicate that PEDV infection can be targeted through multiple antiviral or host-regulatory mechanisms [25–27]. Meanwhile, DS or DS-containing formulations have been reported to improve intestinal health, antioxidant capacity, immune status, and diarrhea-related outcomes in animal or intestinal disease models, and these effects have been linked to pathways such as PI3K/Akt, TLR4/NF-κB, and AMPK/PGC-1α, some of which are closely associated with autophagy regulation [28–30]. Nevertheless, the antiviral effects of DS extract itself and several representative DS-derived bioactive components against PEDV have not been systematically characterized in a unified in vitro model.

Therefore, in the present study, we investigated the antiviral effects of DS extract and its representative bioactive components in PEDV-infected IPEC-J2 cells, with particular attention to pathological injury, viral replication, and autophagy-related gene and protein expression. We further explored whether these effects were associated with modulation of the AMPK/mTOR/autophagy pathway. This study provides experimental evidence to support further evaluation of DS as a potential complementary strategy for the control of PEDV infection.

## Materials and methods

### Materials and reagents

*Codonopsis pilosula* (Dangshen) was purchased from the Tongrentang Pharmacy (Guiyang, China). The five bioactive components of *Codonopsis* including β-sitosterol (SITO), quercetin (Que), 5-hydroxymethylfurfural (HMF), apigenin (APG), and nicotinic acid (NA), were obtained from Macklin Biochemical Technology Co., Ltd (Shanghai, China). Penicillin–streptomycin solution and SDS were purchased from Solarbio (Beijing, China). Fetal bovine serum (FBS, 10%) was obtained from Gibco (South America). The RNAiso Plus reagent kit and SYBR Green Fast qPCR Mix were both sourced from Takara (Japan). The ABScript III RT Master Mix kit was obtained from ABclonal Technology (Wuhan, China). Radioimmunoprecipitation assay (RIPA) buffer and the BCA protein assay kit were purchased from Epizyme Biomedical Technology Co., Ltd (Shanghai, China). Polyvinylidene fluoride (PVDF) membranes were supplied by Millipore (USA). Mouse anti-PEDV N mAb (Catalog No. M100048) was purchased from Zoonogen Co., Ltd. (Beijing, China); LC3 rabbit polyclonal antibody (Catalog No. 14600–1-AP), ATG13 rabbit monoclonal antibody (Catalog No. 82718–3-RR), AMPK rabbit polyclonal antibody (Catalog No. 10929–2-AP), phospho-AMPK rabbit monoclonal antibody (Catalog No. 80209–6-RR) were purchased from Proteintech Group, Inc. (Rosemont, USA); GAPDH Mouse mAb (Catalog No. AC002, source: mouse, monoclonal antibody), β-Actin Rabbit mAb (Catalog No. AC038, source: rabbit, monoclonal antibody) were purchased from ABclonal Biotechnology Co., Ltd. (Wuhan, China). For immunofluorescence staining, FITC-conjugated goat anti-rabbit IgG (GB22303, Wuhan Servicebio Technology Co., Ltd., Wuhan, China) and Cy3-conjugated goat anti-mouse IgG (GB21301, Wuhan Servicebio Technology Co., Ltd., Wuhan, China) were used for the detection of LC3 and PEDV N signals, respectively. The horseradish peroxidase (HRP)-conjugated secondary antibody was obtained from Beyotime Biotechnology (Shanghai, China). The CCK-8 kit was purchased from MedChemExpress (USA).

### Cells and virus

IPEC-J2 porcine intestinal epithelial cells were cultured in DMEM high-glucose medium supplemented with 1% penicillin–streptomycin solution and 10% fetal bovine serum. The IPEC-J2 cell line was obtained from the Laboratory of Basic Veterinary Medicine at Guizhou University.

For viral infection, the IPEC-J2 cells were exposed to the PEDV GDgh strain with the GenBank number of MG983755.1 (kindly provided by Professor Changxu Song from South China Agricultural University) at a multiplicity of infection (MOI) corresponding to 100 TCID_50_. The virus was activated using 5 μg/mL trypsin in DMEM medium. Following infection, the cells were incubated at 37 °C with 5% CO_2_ for 1.5 h to allow viral attachment. Subsequently, the cells were washed with PBS to remove unbound virus, and the infected IPEC-J2 cells were transferred to a fresh DMEM medium for further incubation at different time points.

To determine the virus titer (TCID_50_), IPEC-J2 cells were seeded in a 96-well plate at a density of 1 × 10^5^ cells per well and incubated for 24 h to allow cell attachment. The culture medium was discarded, and cells were washed three times with PBS. The PEDV virus stock was serially diluted in DMEM high-glucose medium (from 10^–1^ to 10^–10^), and 100 µL of each dilution was added to the cells in 96-well plates, with eight replicates per dilution. Negative and positive controls were included, with the negative control containing 2.5 µg/mL trypsin in DMEM and the positive control receiving undiluted virus. After incubating at 37 °C and 5% CO_2_ for 1.5 h for virus adsorption, the virus solution was discarded, and the cells were washed three times with PBS. Each well was then replenished with 100 µL serum-free DMEM, and the cells were incubated at 37 °C and 5% CO_2_ for 72 h. Cytopathic effects (CPE) were observed under a microscope at regular intervals, and the TCID_50_ was calculated using the Reed-Muench method based on the number of wells showing CPE. In this experiment, PEDV infection was carried out in a serum-free DMEM culture medium.

### Preparation of *codonopsis* aqueous extract

*Codonopsis* was extracted using a water decoction method with a material-to-solvent ratio of 1:20 (m:v). The *Codonopsis* root was first crushed into powder and soaked in distilled water for 30 min, followed by boiling for 1 h. The extract was filtered, and the residue was subjected to a second extraction. The filtrates from both extractions were combined and concentrated using a rotary evaporator to a final concentration of 1 g/mL. The concentrated extract was then diluted to 10 mg/mL using DMEM medium and was stored at 4 °C after filtered through a 0.22 μm bacterial filter.

### Cell morphological observation

Cellular morphology was examined using hematoxylin and eosin (H&E) staining and transmission electron microscopy (TEM). For H&E staining, following PEDV infection of IPEC-J2 cells, the cells were harvested at 12, 24, and 48 h post-infection. After removing the culture medium, the cells were washed with PBS. A 4% paraformaldehyde solution was then added to fix the cells at room temperature for 15 min, followed by three washes with PBS to remove excess paraformaldehyde. The cell coverslips were carefully selected and placed with the cell side facing upward on a glass slide. Hematoxylin staining solution was applied for 3 min, followed by differentiation using 1% hydrochloric acid alcohol for several seconds. The slides were then stained with 0.6% ammonia water and eosin solution for 30 s. After drying, the slides were mounted using neutral gum and examined under a microscope to observe the cell morphology.

For TEM analysis, IPEC-J2 cells infected with PEDV were collected at 12, 24, and 48 h post-infection. The cells were washed with PBS and then digested with trypsin to obtain a single-cell suspension. The suspension was centrifuged at 1200 rpm for 5 min, and the supernatant was discarded, retaining the pelleted cells. The cells were fixed in 2% glutaraldehyde (prepared in PBS, 1:5 v/v) for 5 min, followed by incubation in 2% glutaraldehyde at 4 °C for 24 h. After fixation, the cells were further post-fixed in 1% osmium tetroxide for 2 h. The fixed cells were dehydrated in acetone, embedded, and sectioned. Ultrathin sections were placed onto copper grids and stained with uranyl acetate and lead citrate. After washing with water and air drying, the copper grids were examined under a transmission electron microscope. Ultrastructural images were obtained using a JEM-1400FLASH transmission electron microscope (JEOL, Japan) operated at 80 kV and equipped with a Radius imaging system. Images were acquired at magnification of × 20,000.

### RT-qPCR analysis

RT-qPCR was performed to assess the expression levels of autophagy-related genes and the PEDV N gene in IPEC-J2 cells. Published gene sequences were obtained from GenBank, and β-actin was used as the reference gene. Primers were designed and synthesized by Beijing Qingke Biotechnology Co., Ltd., with the relevant primer sequences provided in Table 1. Total RNA from IPEC-J2 cells was extracted using the RNAiso Plus reagent kit, and cDNA was reverse transcribed using the ABScript III RT Master Mix kit. The RT-qPCR reaction volume was 25 μL, consisting of 12.5 μL 2 × SYBR Green Fast qPCR Mix, 1.0 μL of each forward and reverse primer, 2.0 μL cDNA template, and 8.5 μL of ddH2O. The PCR amplification was performed using a two-step protocol: 95 °C for 30 s; 95 °C for 5 s, 59.7 °C for 30 s, for a total of 40 cycles. A melting curve program was set at the end of the amplification. Relative gene expression was calculated using the 2^−ΔΔCt^ method for quantitative analysis.

**Table 1:** Related primer sequence information

Primer name	Primer sequence(5'−3')	Product(bp)	GenBank ID
AMPK	F: GGCAAAGTGAAGGTTGGC	181	NM_001167633.1
	R: CAGATGGTGTACTGATGACC		
mTOR	F: CGTTCATTGGAGATGGTTTG	160	XM_003127584.6
	R: GATGTGGCTTGTTTGATGAG		
LC3	F: ATATCGGAACAGACCTCGTA	95	EU979279.1
	R: TGGCAGGATAAAGTGAAAGG		
ATG13	F: CAGCTTCACAGATTCCTGTA	111	XM_005660951.3
	R: GACAATCACTTGGACAGTCT		
TSC1	F: GCCTCTCGATTACCCACCCT	95	XM_021070814.1
	R: GAAGGCAAAAGAGGTGCTTGG		
TSC2	F: CGTTCTGTCGTGGGAGAGG	187	HQ161901.1
	R: CAGTAGGTGAACTGGCCGTC		
TBC1D7	F: CAGTCAGAGGTTCCCGCTCC	164	XM_005665576.3
	R: CACTGACGAAGCGGATGACG		
Deptor	F: CAATGAGAAATCCCCCGGCT	109	XM_021090557.1
	R: GCTGGGGCTGACTGACATAA		
mLST8	F: ATCTGTACCCGAACCGTA	136	XM_013987831.2
	R: TGATGGGGTTGGGATTATTG		
Tel2	F: GATCTCAATCCGGTCTGC	158	XM_005655139.3
	R: CACGGGAGCCAATAAGTAG		
Tti1	F: CATGGCGGTTTTTGATACTC	185	XM_001925757.5
	R: TCTTCAGGGTAAATCGCAAA		
PEDV N	F: ACTGAACCCACTAACCTGGG	155	MG983755.1
	R: TTGCCATTACCACGACTCCT		
β-actin	F: TCTACACCGCTACCAGTT	126	AY550069.1
	R: ACGATGGAGGGGAAGAC		

### Western blot analysis

After 48 h of PEDV infection, the IPEC-J2 cells were washed with PBS, and total cellular protein was extracted. The cells were lysed with 150 μL of cell lysis buffer containing protease inhibitors and RIPA buffer at a PMSF:RIPA ratio of 1:99, and incubated on ice for 25 min. The lysates were then centrifuged at 13,000 rpm for 15 min at 4 °C, and the supernatant was collected. Protein concentrations were determined using a BCA protein assay kit. The supernatant was mixed with 5 × SDS loading buffer at a 1:5 ratio at 100 °C for 10 min, and was separated using 12.5% SDS-PAGE.

The protein loading in each well was 20 μg, and the membrane transfer conditions were as follows: a constant current of 200 mA was applied on ice, and the transfer time was adjusted according to the size of the protein. The transfer time for β-actin, GAPDH, PEDV N, and LC3 proteins was 30–60 min, and the transfer time for AMPK, p-AMPK, and ATG13 proteins was 60–80 min. Proteins were transferred onto a PVDF membrane, and blocked with 5% non-fat milk, 1% PBS, and 0.1% Tween for 2 h. The membrane was incubated with primary antibodies overnight at 4 °C. The primary antibodies used in this study including anti-PEDV N (1:1000), anti-AMPK (1:1000), anti-LC3 (1:1000), and anti-ATG13 (1:2000). After washing, the membrane was incubated with the appropriate HRP-conjugated secondary antibody (1:1000). Finally, immunoreactive protein bands were visualized using an ECL chemiluminescent substrate.

### Cell activity test

The five active monomeric components of *Codonopsis* were diluted in DMEM medium to a series of concentrations. Cell viability was assessed at 48 h using the CCK-8 assay, with absorbance measured at 450 nm to determine the cell viability levels.

### Immunofluorescence assay

IPEC-J2 cells were fixed overnight in 4% paraformaldehyde solution. After fixation, cells were incubated in PBS containing 0.1% Triton X-100 for 20 min, followed by three washes with PBS. The cells were blocked with 5% non-fat milk at 37 °C for 2 h. Subsequently, the cells were incubated with an anti-PEDV antibody (1:400) at 4 °C overnight and were then incubated with a fluorescently labeled secondary antibody (1:500) at 37 °C for 1 h, followed by three PBS washes. DAPI was added for nuclear counterstaining, and the slides were mounted with an anti-fade reagent. Fluorescence images were captured using an Olympus VS200 fluorescence microscope (Olympus, Japan) with a 20 × objective lens. FITC signals were acquired with excitation at 460–488 nm and emission at 500–530 nm, while Cy3 signals were acquired with excitation at 542–566 nm and emission at 579–611 nm. Images were visualized and analyzed using OlyVIA 4.1 software (Olympus, Japan).

### Statistics and analysis

All experiments were performed in triplicate or more. Western blot and indirect immunofluorescence images were analyzed using ImageJ 5.0 software. Statistical analysis was conducted using SPSS 26.0 software. Prior to one-way ANOVA, normality was assessed separately for each group using the Shapiro–Wilk test, and homogeneity of variance was evaluated using Levene’s test. Differences among groups were analyzed by one-way ANOVA followed by LSD multiple comparisons. Experimental data are presented as mean ± standard deviation (mean ± SD). Graphs were generated using GraphPad Prism 9.0 software. Differences were considered statistically significant at *P* < 0.05, extremely significant at *P* < 0.01, and not significant at *P* > 0.05. For clarity, statistical significance between groups is indicated in the figures using letter notation: uppercase letters (e.g., A, B, C) indicate statistical significance at the *P* < 0.05 level, while lowercase letters (e.g., a, b, c) denote extremely significant differences at the *P* < 0.01 level. Groups marked with the same letter are not significantly different, while groups marked with different letters are significantly different at the respective significance levels.

## Results

### *Codonopsis* extract can mitigate damage to PEDV-induced cellular injury

**Fig. 1:**
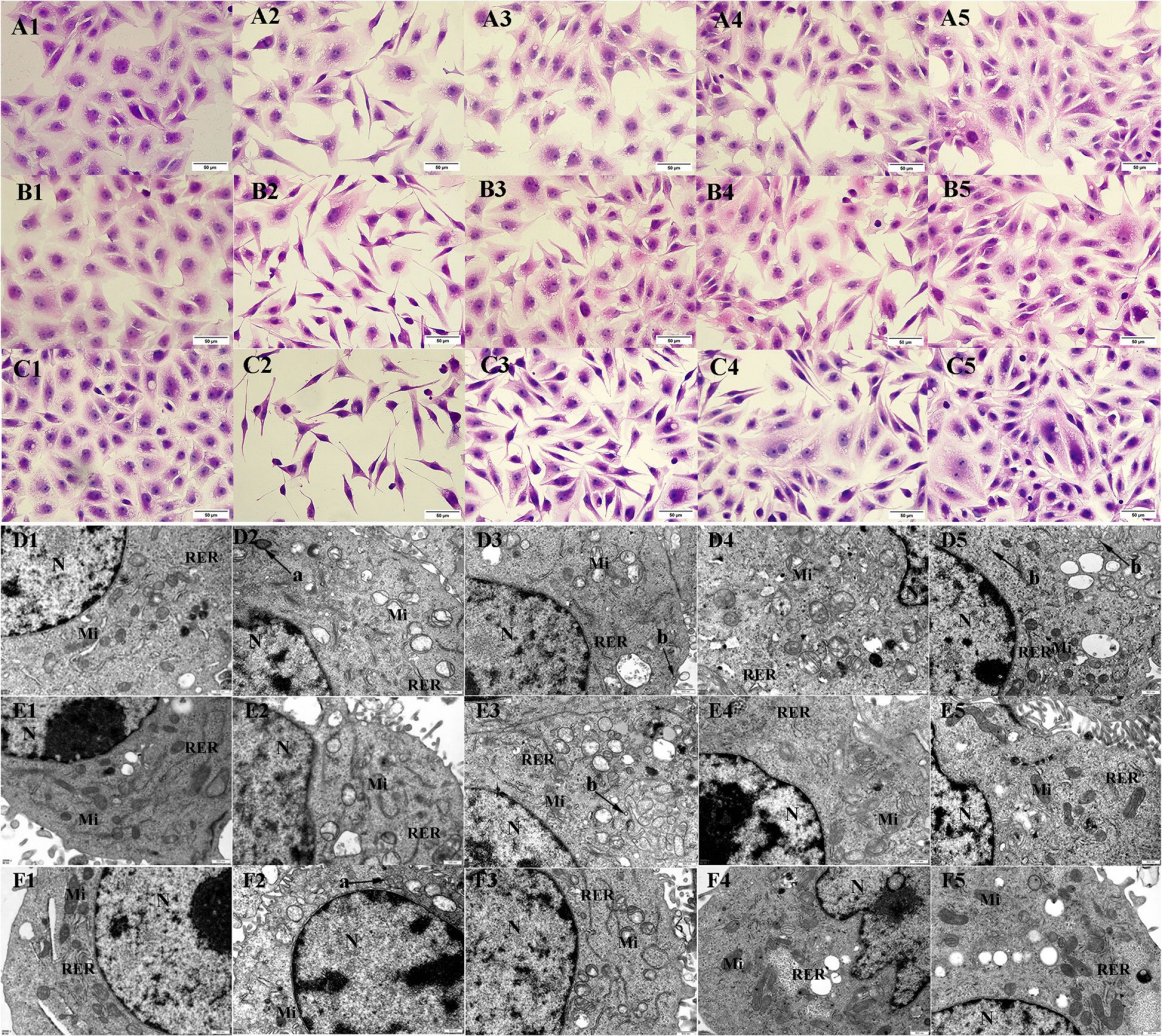
Effects of *Codonopsis* extract on PEDV-infected IPEC-J2 cells as observed by H&E staining (× 400) and transmission electron microscopy (TEM, × 20,000). Notes: Panels **A**, **B**, and **C** represent H&E staining images of cells at 12, 24, and 48 h post-infection, respectively. Panels **D**, **E**, and **F** represent TEM images of cells at 12, 24, and 48 h post-infection, respectively. The numbers 1 ~ 5 following each letter indicate the following experimental groups: negative control group, PEDV-infected group, low-dose *Codonopsis* extract–treated group, medium-dose *Codonopsis* extract–treated group, and high-dose *Codonopsis* extract–treated group, respectively. In the TEM images, “a” denotes autophagosomes, and “b” denotes virus particles. N: Nucleus, Mi: Mitochondria, RER: Rough endoplasmic reticulum

To investigate the effects of *Codonopsis* aqueous extract on PEDV-induced injury in IPEC-J2 cells, cellular morphology was examined using H&E staining and TEM. In the negative group (Fig. 1A1, B1, and C1), cells maintained good growth status at 12, 24, and 48 h, exhibiting regular morphology, predominantly polygonal or short spindle-shaped forms, with dense and uniform arrangement. Cell density gradually increased with prolonged culture time. In contrast, PEDV-infected cells displayed pronounced cytopathic effects at all time points (Fig. 1A2, B2, C2), which progressively worsened over time. By 48 h post-infection, a significant reduction in cell number was observed, with cells becoming slender, spindle-shaped, or irregular in morphology, accompanied by disorganized arrangement and severe disruption of the cell monolayer. In the low-dose (Fig. 1A3, B3, C3) and medium-dose (Fig. 1A4, B4, C4) treated groups, PEDV-induced morphological damage was partially alleviated. At 48 h, both cell number and density were higher than those in the virus-infected group, and cellular morphology appeared more regular; however, complete recovery to normal levels was not achieved. A high dose of *Codonopsis* aqueous extract (Fig. 1A5, B5, C5) markedly attenuated PEDV-induced morphological damage. At 48 h, cellular morphology and growth status closely resembled those of the negative control group.

TEM analysis further supported these observations. Cells in the negative group exhibited intact ultrastructural morphology at 12, 24, and 48 h, with regularly shaped mitochondria, clearly defined cristae, and well-organized rough endoplasmic reticulum (RER). No autophagosomes or autolysosomes were detected (Fig. 1D1, E1, F1). In contrast, PEDV-infected cells (Fig. 1D2, E2, F2) showed pronounced mitochondrial swelling, partial disruption of mitochondrial cristae, and marked dilation of the RER with vesicular changes. Autophagosomes or autolysosomes were observed, and typical viral particles with a diameter of approximately 130 nm were detected beneath the cell membrane. In the low-dose treated group (Fig. 1D3, E3, F3), moderate ultrastructural damage was still evident. Although no obvious autophagosome formation was observed, viral particles remained detectable beneath the cell membrane, suggesting that low-dose Dangshen extract partially alleviated PEDV-induced cellular injury but exerted limited inhibitory effects on viral replication. In the medium-dose group (Fig. 1D4, E4, F4), ultrastructural damage was significantly reduced compared with the infected group. In the high-dose group (Fig. 1D5, E5, F5), the overall ultrastructural morphology of cells was close to normal, with no detectable autophagosomes and substantially reduced organelle damage compared with other treatment groups. Observation of the ultrastructural images showed that PEDV infection caused marked damage mainly to the rough endoplasmic reticulum and mitochondria in IPEC-J2 cells. In contrast, treatment with *Codonopsis pilosula* extract alleviated the virus-induced ultrastructural damage to these organelles.

Accordingly, PEDV infection severely disrupts normal cellular morphology and growth, whereas *Codonopsis* aqueous extract alleviates PEDV-induced cellular injury and improves cellular structural integrity in a dose-dependent manner. Notably, high-dose *Codonopsis* extract provided the most pronounced protective effect against PEDV infection in IPEC-J2 cells by effectively inhibiting autophagosome formation and mitigating pathological damage.

### *Codonopsis* extract attenuates PEDV-induced autophagy

**Fig. 2:**
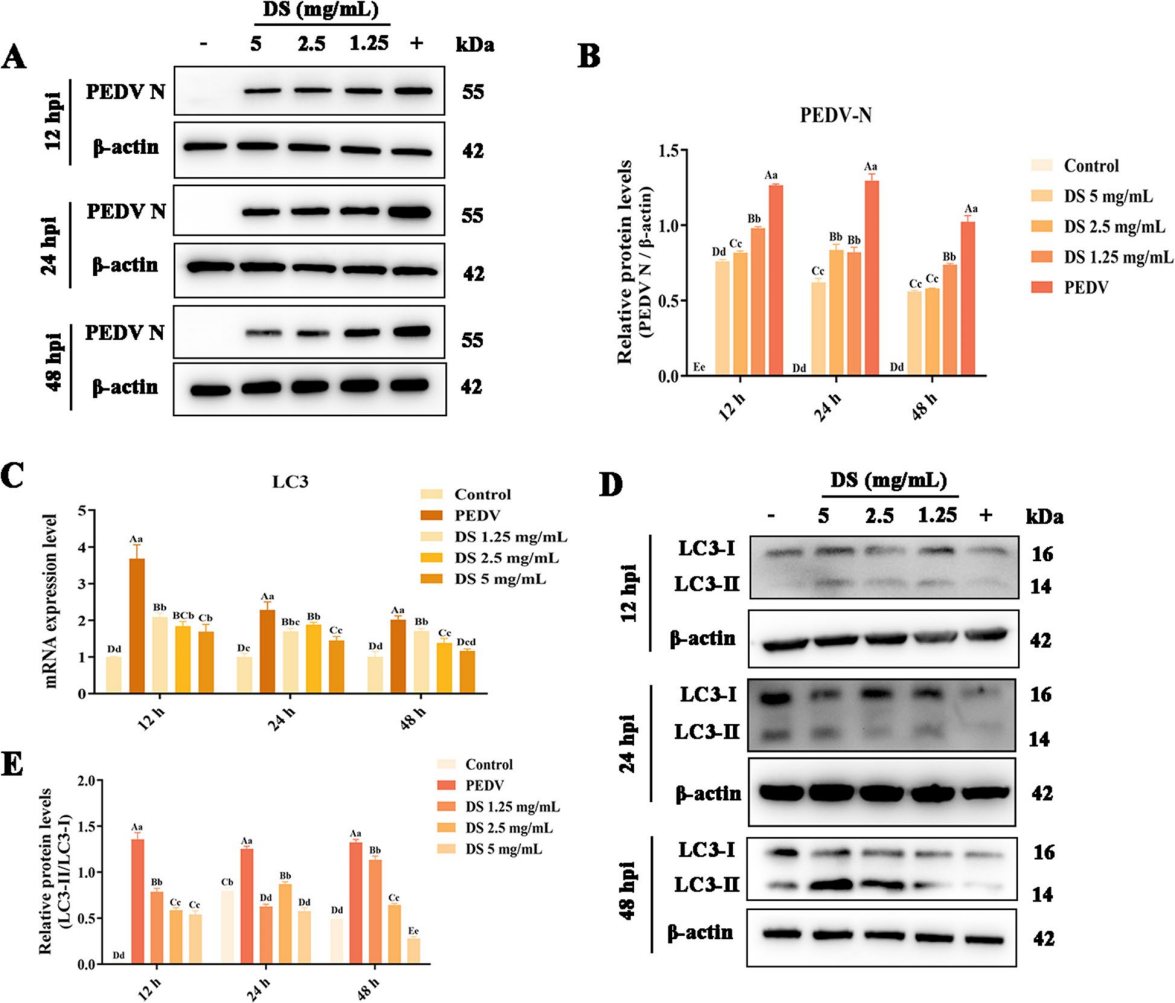
The effects of *Codonopsis* extract on the autophagy of PEDV-infected IPEC-J2 cells. Western blotting results of β-actin and PEDV N in PEC-J2 cells (**A**), the quantification of PEDV N/β-actin (**B**), the mRNA expression levels of LC3 gene (**C**), western blotting results of β-actin, LC3- I and LC3-II (**D**), and the quantification of LC3-II/LC3I (**E**). Different uppercase letters (e.g., **A**, **B**, **C**) indicate significant differences among groups (*P* < 0.05), while different lowercase letters (e.g., a, b, c) indicate extremely significant differences (*P* < 0.01). Values sharing at least one identical letter are not significantly different. The value labeled “Aa” represents the highest level among groups. Notes: DS, Dangshen (*Codonopsis*)

To investigate whether *Codonopsis* aqueous extract alleviates PEDV-induced cellular injury by suppressing autophagy, Western blot analysis was first performed to examine the expression of the PEDV N protein in IPEC-J2 cells. The results showed that no PEDV N protein was detected in the negative group at 12, 24, or 48 h, whereas PEDV N protein expression was dramatically increased in the infected group. Compared with the infected group, treatment with high-, medium-, and low-dose *Codonopsis* aqueous extract significantly inhibited PEDV N protein expression (*P* < 0.01), exhibiting an overall dose-dependent trend. At 12 and 24 h, the inhibitory effect of high-dose group on PEDV N protein expression was significantly stronger than that of the medium- and low-dose groups (*P* < 0.01). At 48 h, no significant difference was observed between the high- and medium-dose groups in suppressing PEDV N protein expression (*P* > 0.05); however, both groups showed significantly greater inhibition than the low-dose group (*P* < 0.01) (Fig. 2A and B).

Based on the confirmed antiviral activity of *Codonopsis* aqueous extract against PEDV, its potential protective role via modulation of autophagy was further explored. RT-qPCR analysis revealed that *LC3* mRNA expression levels in IPEC-J2 cells were significantly higher than those in the negative control group at 12, 24, and 48 h (*P* < 0.01), indicating that PEDV infection markedly induced autophagy. At 12 h, *LC3* mRNA expression in the high-dose group did not differ significantly from that in the medium-dose group (*P* > 0.05) but was significantly lower than that in the low-dose group (*P* < 0.05). At 24 h, *LC3* mRNA expression was further reduced in all treated groups. At 48 h, *LC3* mRNA expression in the high-dose group was approaching to the negative group, whereas the low- and medium-dose groups maintained relatively higher expression levels, demonstrating a dose-dependent effect (Fig. 2C).

The regulatory effect of *Codonopsis* aqueous extract on autophagy was further validated at the protein level. Western blot analysis showed that LC3-II/I protein expression in IPEC-J2 cells was significantly higher than that in the negative group at 12, 24, and 48 h (*P* < 0.01), indicating robust activation of autophagy following PEDV infection. Compared with the infected group, treatment with high-, medium-, and low-dose *Codonopsis* aqueous extract significantly reduced LC3-II/I protein expression levels (*P* < 0.01). However, no consistent time-dependent pattern was observed in the medium- and low-dose groups. At 48 h, suppression of LC3-II/I protein expression by *Codonopsis* aqueous extract exhibited a pronounced dose-dependent effect, with the high-dose group showing the most significant inhibition (Fig. 2D and E).

### The effects of *Codonopsis* extract on AMPK/mTOR pathway-related genes and proteins

To further elucidate whether the inhibitory effect of *Codonopsis* aqueous extract on PEDV-induced autophagy is associated with regulation of the AMPK/mTOR signaling pathway, RT-qPCR analysis was performed at 48 h post-infection to assess the mRNA expression levels of autophagy-related genes, including *AMPK*, *mTOR*, and *ATG13* mRNA, in IPEC-J2 cells.

**Fig. 3:**
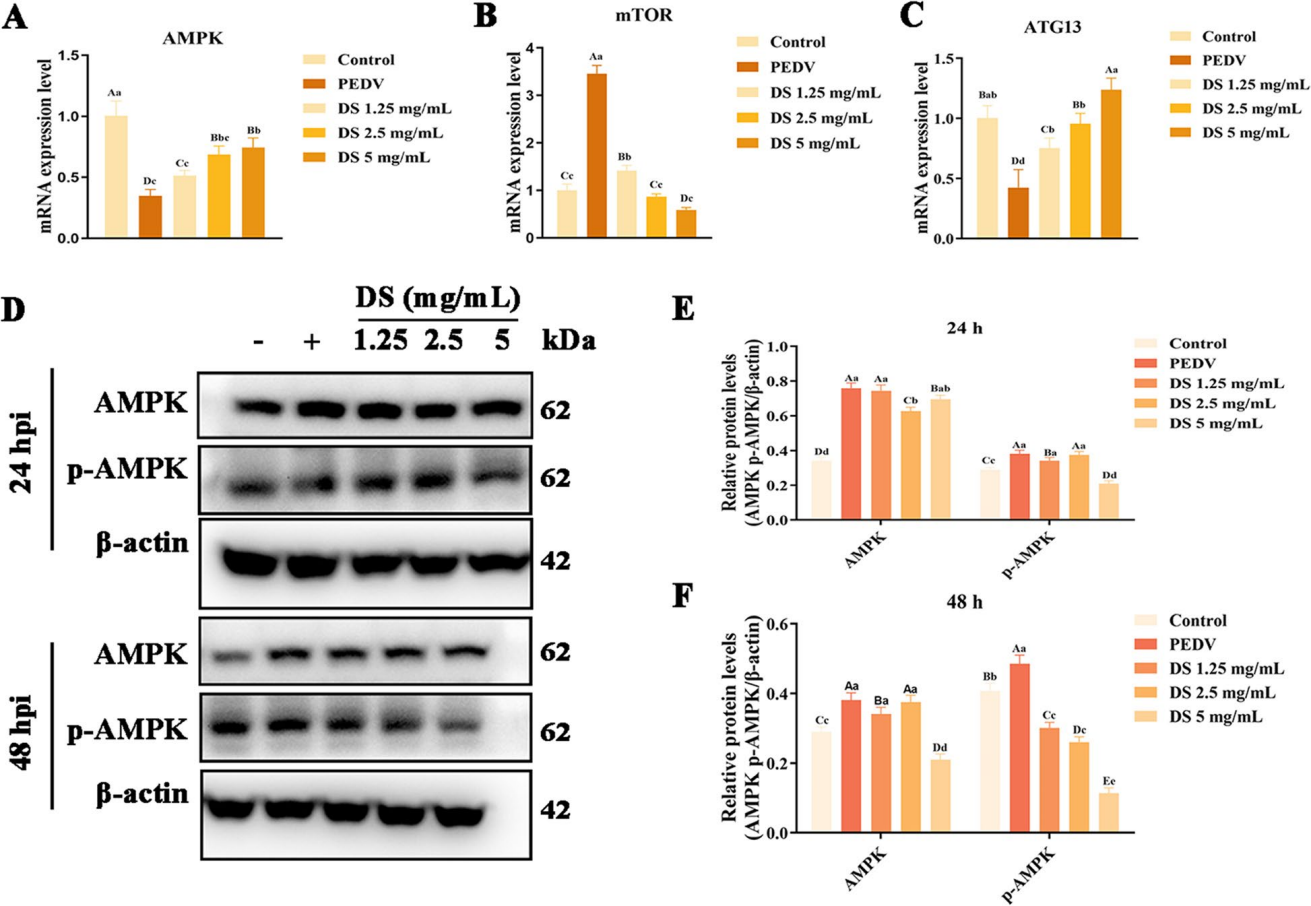
The effects of *Codonopsis* extract on AMPK/mTOR pathway-related genes and proteins. The mRNA expression levels of *AMPK* (**A**), *mTOR* (**B**), and *ATG13* (**C**); Western blotting results of AMPK and p-AMPK in PEC-J2 cells (**D**); the quantification of AMPK/β-actin and p-AMPK/β-actin at 24 h (**E**) and at 48 h (**F**). Different uppercase letters (e.g., **A**, **B**, **C**) indicate significant differences among groups (*P* < 0.05), while different lowercase letters (e.g., a, b, c) indicate extremely significant differences (*P* < 0.01). Values sharing at least one identical letter are not significantly different. The value labeled “Aa” represents the highest level among groups. Notes: DS, Dangshen (*Codonopsis*)

The results showed that (Fig. 3A, B and C), compared with the negative control group, PEDV infection significantly downregulated the expression of *AMPK* and *ATG13* mRNA, while markedly upregulating *mTOR* mRNA expression in IPEC-J2 cells (*P* < 0.01), indicating that PEDV infection profoundly disrupts the transcriptional regulation of the AMPK/mTOR signaling pathway. In contrast, treatment with *Codonopsis* aqueous extract resulted in varying degrees of upregulation of *AMPK* and *ATG13* mRNA expression compared with the infected group, with a clear dose-dependent pattern. The expression levels of *AMPK* and *ATG13* mRNA in the high-dose group were significantly higher than those in the low-dose group (*P* < 0.01). Meanwhile, *Codonopsis* aqueous extract significantly suppressed the aberrant upregulation of *mTOR* mRNA induced by PEDV infection (*P* < 0.01), with the strongest inhibitory effect observed in the high-dose group, followed by the medium-dose group.

The expression of AMPK and its phosphorylated form (p-AMPK) was further analyzed by Western blotting. The results (Fig. 3D and E) demonstrated that PEDV infection significantly increased AMPK and p-AMPK protein levels in IPEC-J2 cells at 24 h post-infection (*P* < 0.01). Treatment with medium- and high-dose *Codonopsis* aqueous extract significantly reduced AMPK protein levels (*P* < 0.05), whereas no significant difference was observed between the low-dose group and the infected group (*P* > 0.05). At 48 h post-infection, *Codonopsis* aqueous extract treatment markedly reduced p-AMPK protein levels (*P* < 0.01), with the high-dose group exhibiting strongest inhibitory effect (Fig. 3F). These findings indicate that *Codonopsis* aqueous extract effectively suppresses PEDV-induced excessive phosphorylation of AMPK.

### Safety evaluation of the active components of *Codonopsis*

**Fig. 4:**
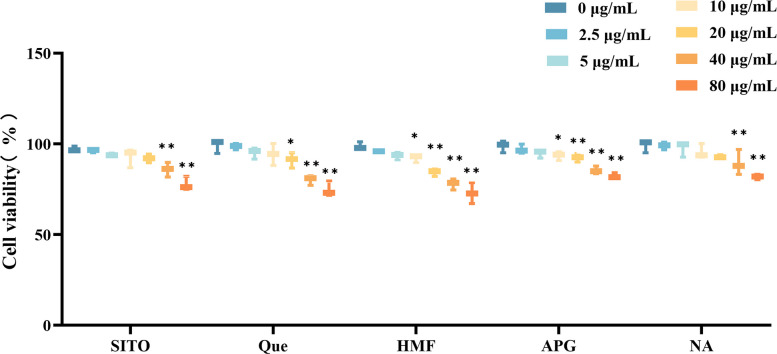
Safety assessment of five active components of *Codonopsis* in IPEC-J2 cells. The values are expressed as the means ± SDs of triplicate values. *P < 0.05 and **P < 0.01, compared with the blank control group. Notes: SITO, β-sitosterol; Que, quercetin; HMF, 5-hydroxymethylfurfural; APG, apigenin; NA, nicotinic acid

To determine the in vitro cytotoxicity of the five major bioactive components of *Codonopsis* and to identify appropriate concentrations, the cytotoxic effects of SITO, QUE, HMF, APG, and NA on IPEC-J2 cells were evaluated. As shown in Fig. 4, compared with the blank control group, SITO and NA significantly reduced IPEC-J2 cell viability at a concentration of 40 μg/mL (*P* < 0.01). Que significantly inhibited cell viability at 20 μg/mL (*P* < 0.05). Besides, IPEC-J2 cells were more sensitive to HMF and APG, with a significant decrease in cell viability observed at 10 μg/mL (*P* < 0.05). Accordingly, based on the principle of selecting the maximum non-cytotoxic dose prior to a significant reduction in cell viability, the following working concentrations were chosen for subsequent in vitro experiments: 20 μg/mL for SITO and NA, 20 μg/mL for Que, and 5 μg/mL for HMF and APG. These concentrations maintained stable cell viability while minimizing potential interference from compound-induced cytotoxicity.

### The effects of five active components of *Codonopsis* on autophagy of PEDV-infected IPEC-J2 cells

At 48 h post-infection, this study systematically compared the regulatory effects of five individual bioactive components of *Codonopsis* on PEDV replication and the cell autophagic responses to elucidate their potential antiviral mechanisms.

**Fig. 5:**
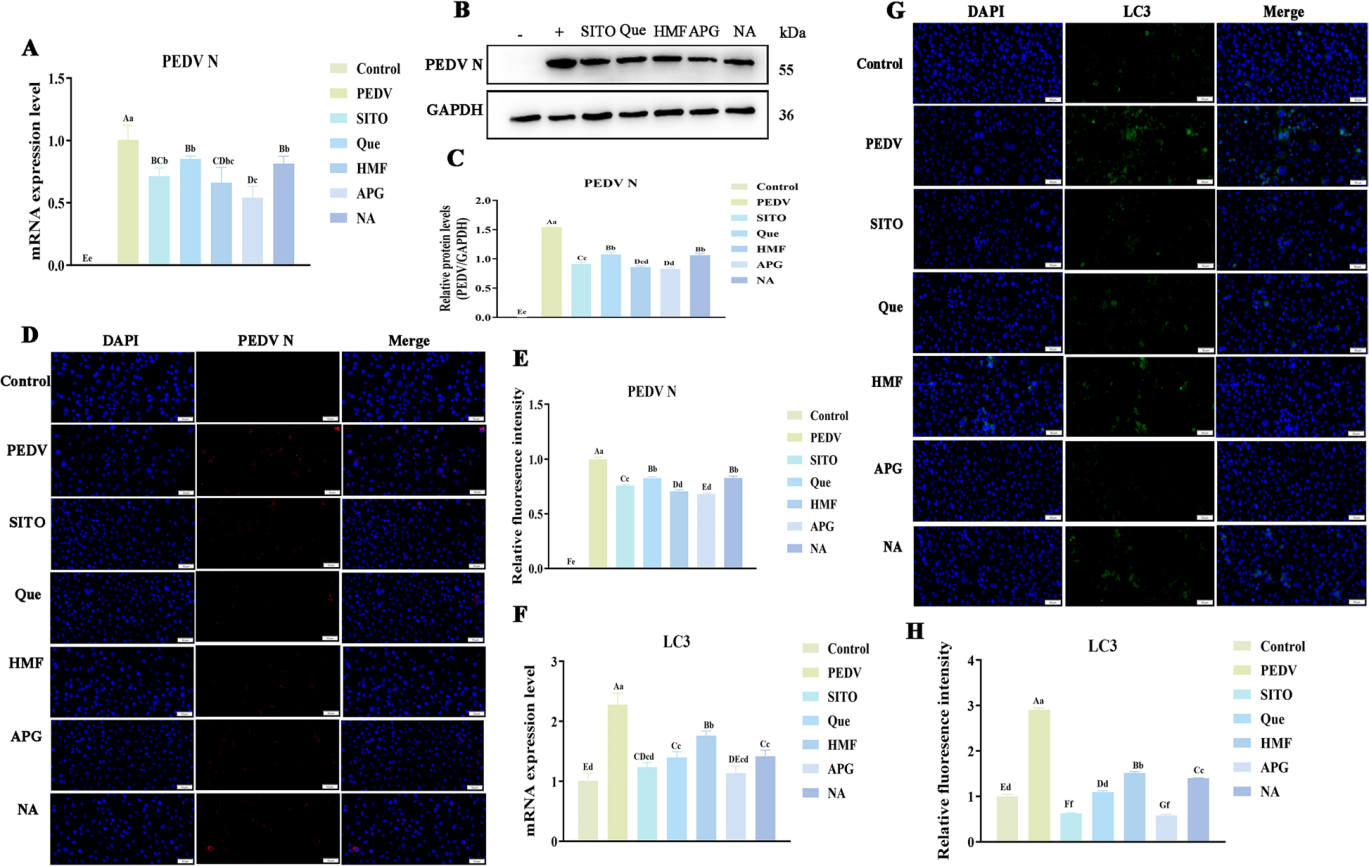
The effects of five active components of *Codonopsis* on autophagy of PEDV-infected IPEC-J2 cells. The mRNA expression levels of *PEDV N* gene (**A**), western blotting results of GAPDH and PEDV N (**B**), the quantification of PEDV N/GAPDH (**C**), the indirect immunofluorescence assays of PEDV N (**D**), and relative fluorescence of PEDV N (**E**), the mRNA expression levels of *LC3* gene (**F**), the indirect immunofluorescence assays of LC3 (**G**), and relative fluorescence of LC3 (**H**). Different uppercase letters (e.g., **A**, **B**, **C**) indicate significant differences among groups (*P* < 0.05), while different lowercase letters (e.g., a, b, c) indicate extremely significant differences (*P* < 0.01). Values sharing at least one identical letter are not significantly different. Notes: SITO, β-sitosterol; Que, quercetin; HMF, 5-hydroxymethylfurfural; APG, apigenin; NA, nicotinic acid

RT-qPCR analysis showed that *PEDV N* mRNA was undetectable in the negative control group, whereas its expression was markedly elevated in the infected group (Fig. 5A). Compared with the infected group, treatment with each of the five *Codonopsis* bioactive components resulted in a highly significant downregulation of *PEDV N* mRNA expression (*P* < 0.01). Among these compounds, APG exhibited the strongest inhibitory effect on *PEDV N* mRNA expression, followed by HMF, SITO, NA, and Que. At the protein level, Western blot analysis yielded results highly consistent with the transcriptional data (Fig. 5B and C). At 48 h post-infection, five bioactive components significantly suppressed PEDV N protein expression compared with the infected group *(P* < 0.01), and the relative inhibitory potency closely mirrored the changes observed at the mRNA level.

To further visually confirm the inhibitory effects of these bioactive components on PEDV replication, indirect immunofluorescence assays were performed to assess intracellular PEDV N protein expression (Fig. 5D and E). The results demonstrated that, compared with the infected group, all five bioactive components significantly reduced the fluorescence intensity of PEDV N protein (*P* < 0.01). The inhibitory efficacy ranked from strongest to weakest as follows: APG > HMF > SITO > QUE ≈ NA, thereby corroborating the molecular findings at the cellular level.

Analysis of autophagy-related indicators revealed that PEDV infection markedly upregulated *LC3* mRNA expression in IPEC-J2 cells (*P* < 0.01) (Fig. 5F). In contrast, treatment with each of the five Dangshen bioactive components significantly reduced *LC3* mRNA expression compared with the infected group (*P* < 0.01). Results of indirect immunofluorescence assays was consistent with that of the *LC3* mRNA expression (Fig. 5G and H). The inhibitory effects of five active components of *Codonopsis* on LC3 protein followed the order: APG > SITO > QUE > NA > HMF. When integrated with the results on PEDV N protein, APG consistently exhibited the strongest inhibitory effects on both PEDV replication and LC3 expressions. These results suggest that the anti-PEDV activity of APG is closely associated with its effective modulation of virus-induced autophagy.

### The effects of five bioactive components of *codonopsis* on AMPK/mTOR pathway related genes and proteins

**Fig. 6:**
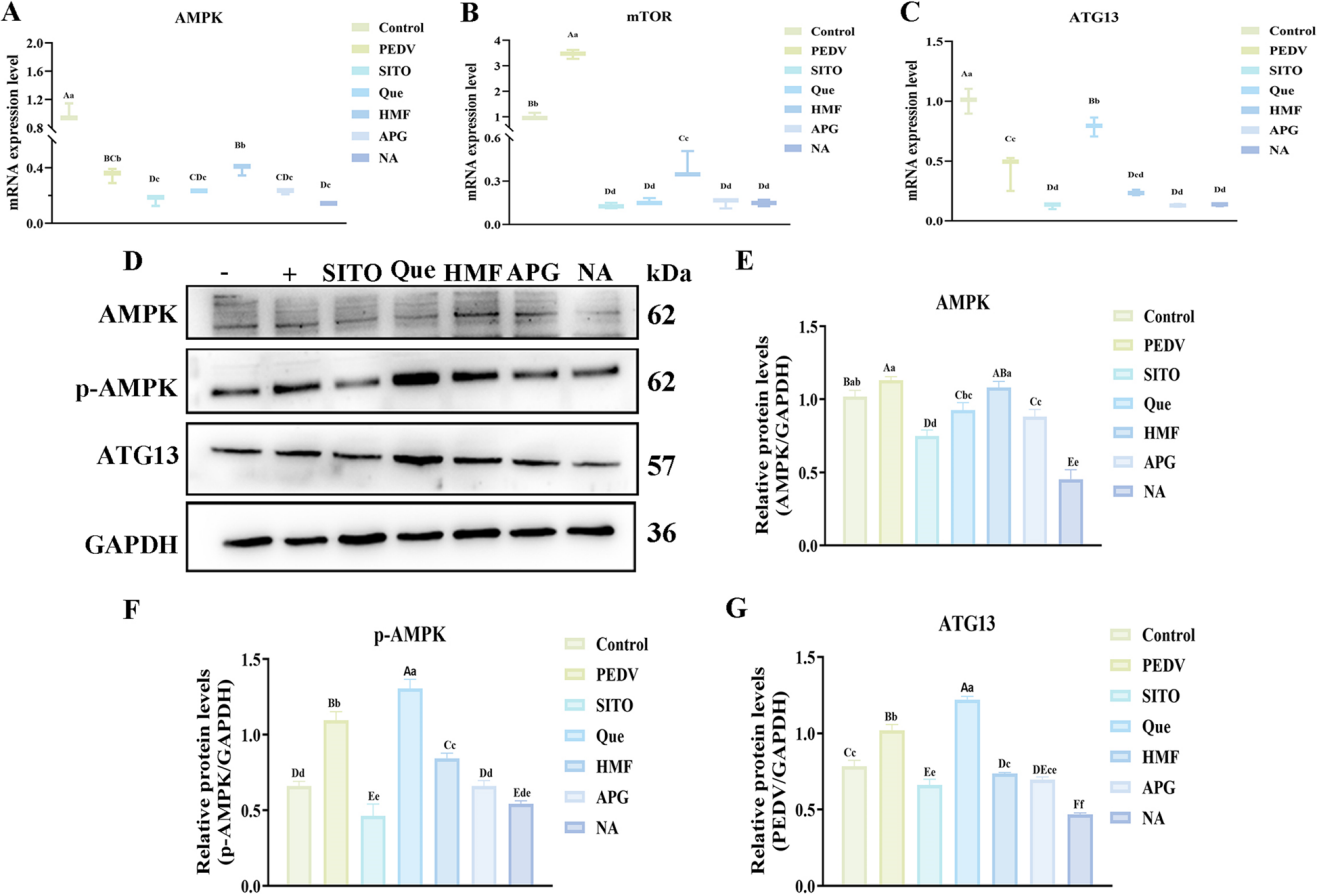
The effects of five bioactive components of *codonopsis* on AMPK/mTOR pathway related genes and proteins. The mRNA expression levels of *AMPK* (**A**), *mTOR* (**B**), and *ATG13* (**C**); western blotting results of AMPK, p-AMPK and ATG13 (**D**); the quantification of AMPK/GAPDH (**E**), p-AMPK/GAPDH (**F**), and ATG13/GAPDH (**G**). Different uppercase letters (e.g., **A**, **B**, **C**) indicate significant differences among groups (*P* < 0.05), while different lowercase letters (e.g., a, b, c) indicate extremely significant differences (*P* < 0.01). Values sharing at least one identical letter are not significantly different. Notes: SITO, β-sitosterol; Que, quercetin; HMF, 5-hydroxymethylfurfural; APG, apigenin; NA, nicotinic acid

Compared with the negative control group, PEDV infection markedly downregulated the expression of *AMPK* and *ATG13* mRNA in IPEC-J2 cells, while significantly upregulating *mTOR* mRNA expression (*P* < 0.01) (Fig. 6A, B and C). Compared with the infected group, treatment with the bioactive components SITO, QUE, APG, and NA further reduced *AMPK* mRNA expression. The QUE increased *ATG13* mRNA levels, whereas the other four bioactive components significantly decreased *ATG13* mRNA expression. These results are inconsistent with the dose-dependent recovery of *AMPK* and *ATG13* mRNA expression observed following treatment with *codonopsis* aqueous extract, indicating substantial differences between the whole extract and individual components in transcriptional regulation. Notably, despite the different effects of the bioactive components and the aqueous extract on *AMPK* and *ATG13* mRNA transcription, all five bioactive components significantly suppressed the PEDV-induced abnormal upregulation of *mTOR* mRNA (*P* < 0.01). The inhibition of bioactive components was generally greater than that observed with the *Codonopsis* aqueous extract. Except for HMF, no significant differences were observed among the remaining four components in their inhibitory effects on *mTOR* mRNA expression.

At the protein level, western blot analysis showed that PEDV infection significantly increased the expression of AMPK, p-AMPK, and ATG13 in IPEC-J2 cells compared with the negative control group (*P* < 0.05) (Fig. 6D). Compared with the infected group, SITO, Que, APG, and NA significantly reduced AMPK protein expression (*P* < 0.01), with AMPK levels in some treatment groups even falling below those of the negative control group (Fig. 6E). In addition, Que significantly increased the protein expression of p-AMPK and ATG13 relative to the infected group (*P* < 0.01), whereas the other four bioactive components markedly suppressed the expression of p-AMPK and ATG13 (*P* < 0.01) (Fig. 6F and G).

## Discussion

*Codonopsis pilosula*, a traditional Chinese medicinal herb, has attracted increasing attention due to its favorable safety profile and its diverse pharmacological activities, including anti-inflammatory, immunomodulatory, and gastrointestinal mucosal protective effects [31]. Because PEDV primarily targets intestinal epithelial cells, leading to severe disruption of the intestinal mucosal barrier and profuse diarrhea, there is practical value in developing candidate interventions that combine inhibition of viral replication with preservation of epithelial barrier integrity. PEDV continues to circulate widely and undergo frequent genetic variation, and PEDV possesses multiple mechanisms of innate immune evasion, resulting in the convergence of impaired host antiviral responses and exacerbated tissue injury. These features further underscore the importance of multitarget strategies based on natural products [32]. Therefore, the present study systematically evaluated the antiviral effects of *codonopsis* aqueous extract and its major bioactive components in a PEDV-infected IPEC-J2 cell model.

Previous studies have demonstrated that PEDV infection induces pronounced structural damage to small intestinal epithelial cells, including microvillus shortening, mitochondrial swelling, and endoplasmic reticulum dilation [33]. At present, there are no specific therapeutic agents available for PEDV, and vaccines provide limited protection against variant strains, highlighting the urgent need to develop novel anti-PEDV strategies. In recent years, many research has focused on the potential of natural products and active components of traditional Chinese medicine as anti-PEDV agents. A variety of plant extracts, vitamin derivatives, and natural small molecules have been reported to inhibit PEDV replication or relieve virus-induced cellular injury [34–36]. For example, hyperoside isolated from hawthorn has been shown to exert anti-PEDV activity both in vitro and in vivo by inhibiting the interaction between PEDV N protein [37]. Levoglucosan A, a natural compound, also exhibits antiviral activity against PEDV, inhibiting viral replication in a dose-dependent manner with enhanced efficacy over time [38]. Fangchinoline suppresses PEDV by interfering with viral attachment, internalization, and replication, resulting in dose-dependent restriction of PEDV replication in IPEC-J2 cells [39]. Glycyrrhizin, a major constituent of licorice, inhibits PEDV proliferation and reduces proinflammatory cytokine production by modulating the HMGB1/TLR4–p38 signaling pathway [40]. In this study, morphological analysis by H&E staining and TEM revealed that PEDV infection markedly exacerbated cellular injury in IPEC-J2 cells. In contrast, treatment with *codonopsis* aqueous extract substantially reduced cell shrinkage, rounding, and detachment, while significantly alleviating mitochondrial and rough endoplasmic reticulum damage. The protective effect was most pronounced in the high-dose *codonopsis* treatment group.

Autophagy, as a critical regulatory mechanism by which host cells respond to viral infection, plays a dual role during PEDV infection. It is reported that PEDV can induce autophagy through activation of the AMPK signaling pathway, thereby creating a cellular environment favorable for viral replication [9]. Conversely, under certain conditions, activation of autophagy may also restrict viral propagation and attenuate virus-induced cell death [41] . In this study, PEDV infection promoted the conversion of LC3-I to LC3-II, and the formation of autophagosomes accompanied by the accumulation of viral particles was observed by TEM. These findings indicate that PEDV induces autophagic responses in IPEC-J2 cells. Compared with the PEDV-infected group, treatment with *codonopsis* aqueous extract significantly downregulated both the gene and protein expression levels of LC3 and reduced autophagosome formation. This observation is consistent with previous reports demonstrating that suppression of autophagy through key regulatory nodes such as Beclin1 and ATG5 can modulate inflammatory pathways and alleviate virus-associated cellular damage [42]. Moreover, *codonopsis* aqueous extract can reduce PEDV N protein expression and suppressed PEDV-induced autophagy by upregulating *mTOR* mRNA expression while downregulating *AMPK* and *ATG13* mRNA level, as well as inhibiting AMPK protein expression and its phosphorylation.

Mechanistically, these changes can be interpreted as a coordinated shift of the AMPK/mTOR/ULK1–ATG13 axis toward a less autophagy-permissive state. In PEDV-infected cells, AMPK activation has been shown to promote autophagy and viral replication, and AMPK inhibition suppresses both processes [9]. At the signaling level, AMPK can facilitate autophagy initiation by enhancing ULK1 activity and, under energy stress conditions, by inhibiting mTORC1 signaling (e.g., through raptor-dependent mechanisms) [43, 44]. Conversely, mTORC1 is a key negative regulator of autophagy initiation and directly regulates the ULK1–ATG13–FIP200 complex, which serves as a central integration node for upstream nutrient/energy signals [44–46]. Therefore, in the present study, suppression of p-AMPK likely weakens the pro-autophagic drive, whereas increased mTOR mRNA suggests partial restoration of host anabolic/homeostatic signaling and a trend toward enhanced anti-autophagic restraint. These two regulatory directions are not contradictory; rather, they are synergistic in predicting reduced autophagy initiation, which is consistent with the observed decreases in LC3 expression and autophagosome formation.

**Fig. 7:**
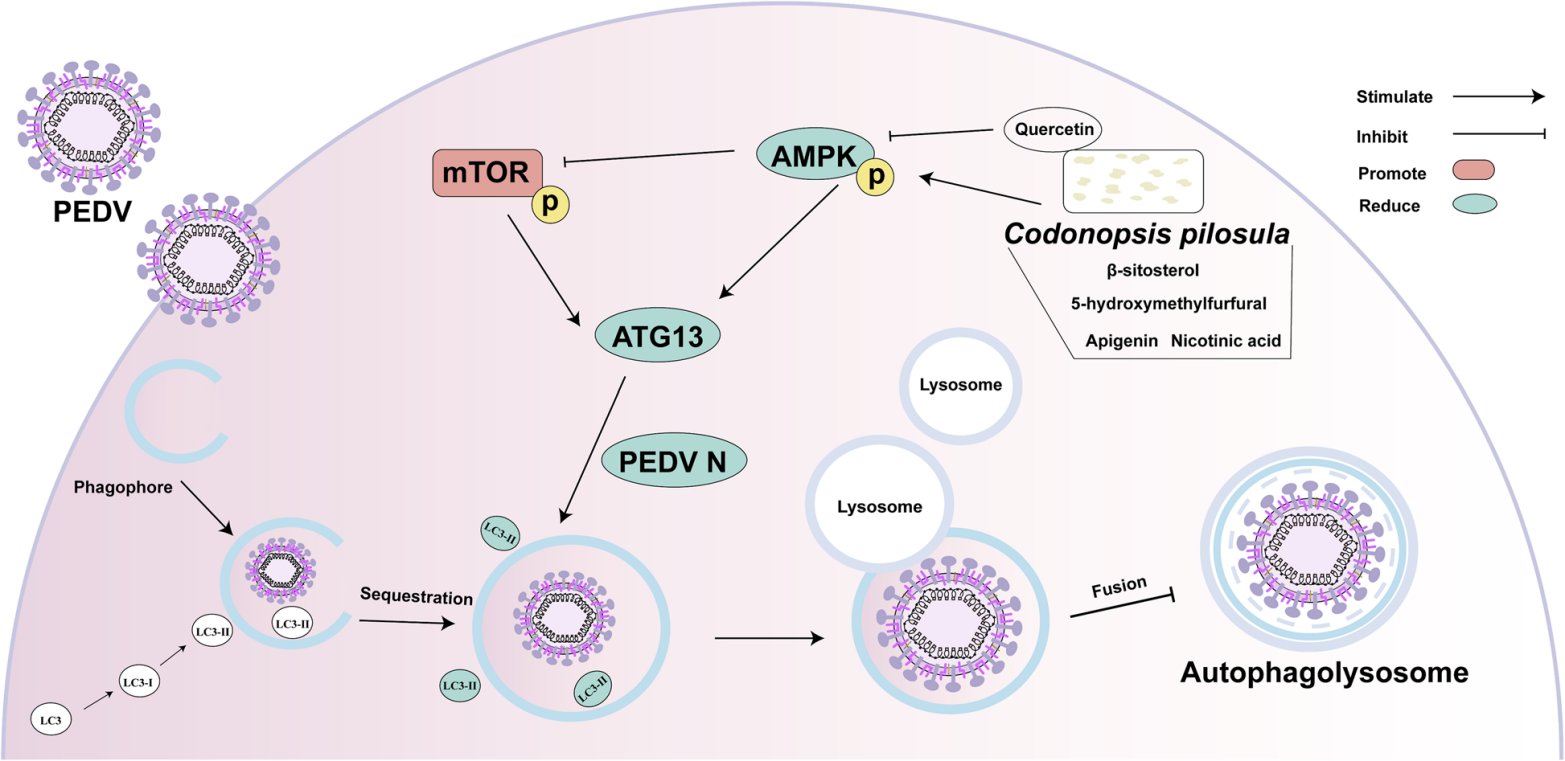
Mechanism of *codonopsis* extract and bioactive components in alleviating cell autophagy induced by EPDV via the AMPK/mTOR pathway

At the same time, it should be noted that increased mTOR mRNA does not necessarily equate to increased mTORC1 kinase activity, which is more directly reflected by phosphorylation readouts and downstream substrates. Thus, in this study, the inference of autophagy suppression is strengthened not by mTOR mRNA alone, but by the convergence of multiple observations (reduced p-AMPK/AMPK protein, reduced LC3, fewer autophagosomes, and reduced PEDV N protein). This “convergent evidence” interpretation is particularly important in virology studies, because PEDV-related autophagy can be stage-dependent and flux-incomplete (e.g., autophagosome accumulation with impaired autolysosome formation has also been reported) [47]. Thus, these regulatory effects converge to attenuate PEDV-induced autophagic activity (Fig. 7).

While autophagy was not directly manipulated in this study (e.g., through the use of inducers, inhibitors, or genetic knockdown), convergent evidence from viral titer, N protein levels, autophagosome counts, and pathway phosphorylation robustly supports the link between antiviral activity and autophagy inhibition. Future studies employing direct manipulation of autophagy will be crucial to further validate this relationship and elucidate the precise mechanisms involved.

The effects of individual bioactive components of *codonopsis* on PEDV replication and on *LC3* mRNA and protein expression were largely consistent with those observed for the aqueous extract. Numerous active constituents derived from traditional Chinese medicine are known to directly interfere with specific stages of the viral life cycle, thereby preventing viral replication. For example, panax notoginseng saponin has been reported to directly suppress PEDV replication in infected Vero cells in a dose-dependent manner [48]. Among bioactive components of *codonopsis*, apigenin (APG) exhibited the strongest inhibitory effects on *PEDV N* mRNA and protein expression, followed by 5-hydroxymethylfurfural (HMF), β-sitosterol (SITO), nicotinic acid (NA), and quercetin (Que). Notably, APG not only significantly reduced *PEDV N* mRNA and protein expression but also downregulated multiple autophagy-related genes and proteins within the AMPK/mTOR signaling pathway. Its inhibitory efficacy was greater than that of the other tested components, suggesting that APG may be a key component underlying the anti-PEDV activity of *codonopsis*. APG is a flavonoid compound characterized by low aqueous solubility and high membrane permeability, enabling efficient translocation across host cell membranes. Previous studies have demonstrated that APG can enhance autophagosome formation and promote LC3-I to LC3-II conversion in HepG2 cells, thereby facilitating lipid metabolism through activation of the AMPK signaling pathway [49].

Notably, *codonopsis* aqueous extract and its individual bioactive components exhibited distinct regulatory patterns within the AMPK/mTOR signal pathway. As a multicomponent system, the aqueous extract exhibited a recovery regulation on *AMPK* and *ATG13* mRNA transcription, characterized by a clear dose-dependent trend. In contrast, except that quercetin restored *ATG13* mRNA expression, most individual bioactive components suppressed *AMPK* and *ATG13* mRNA levels. Five monomeric compounds can significantly suppress *mTOR* mRNA levels, with nicotinic acid showing the strongest suppression. These results likely reflect differences in antiviral mechanisms between the aqueous extract and individual compounds. First, *codonopsis* aqueous extract contains a complex mixture of polysaccharides, fructans, phenolic acids, and flavonoids. Such multicomponent formulations are more effective at modulating the “inflammatory microenvironment–cellular homeostasis” axis rather than strong single target. By contrast, the five monomeric compounds are more likely to exert antiviral effects through direct and potent suppression of viral replication, manifested by strong inhibition of aberrant *mTOR* mRNA expression and the obvious downregulation of AMPK, p-AMPK and ATG13 protein expression. Besides, the bioactive monomers were administered at their maximum non-cytotoxic concentrations, resulting in higher exposure than that of corresponding constituents present within the aqueous extract. This exposure may account for the stronger suppression of key regulatory genes (*AMPK* and *ATG13* mRNA expression). The multiple constituents within the aqueous extract may lead to synergistic or antagonistic interactions that could buffer the excessive inhibition of individual targets. In this study, the QUE showed different effects from other components on protein expression suggests that individual components may had opposing regulatory influences on the same signal element. Furthermore, the antiviral activity of *codonopsis* aqueous extract likely involves multitarget actions, which is consistent with multitarget antiviral mechanisms characteristic of natural products [50].

Importantly, the present study does not formally prove synergy or antagonism among defined *codonopsis* constituents, because combination effects were not quantified using dose–response matrix designs and synergy models. Therefore, the concept of “multi-component synergy” is introduced here as a plausible mechanistic framework to interpret the extract–monomer divergence, rather than as a definitive conclusion. Future studies should combine fractionation–recombination strategies with quantitative combination analyses (e.g., Loewe/Bliss/HSA/ZIP frameworks) and pathway-specific readouts to determine whether the ATG13 divergence reflects true synergy, antagonistic buffering, or concentration/time-dependent network compensation [51, 52].

Accordingly, *codonopsis* aqueous extract attenuates PEDV replication and corrects virus-induced AMPK hyperactivation and aberrant *mTOR* mRNA expression, thereby reducing LC3-associated autophagy and alleviating organelle damage. In contrast, owing to higher effective intracellular exposure, individual bioactive components exert stronger suppression of *mTOR* mRNA expression and phosphorylation networks. However, both the extract and its monomeric components ultimately converge on the AMPK/mTOR autophagy pathway to inhibit PEDV replication.

Despite these findings, several limitations of the present study should be considered. This study was based on a single intestinal epithelial cell line, IPEC-J2, which inevitably limits the broader applicability of the results. Moreover, all experiments were conducted in vitro, and no in vivo or ex vivo models were used to validate the protective effect of *Codonopsis pilosula* extract under more physiologically relevant conditions. Another limitation is that only one PEDV strain was tested, although strain-to-strain variation may influence both viral pathogenicity and host-cell responses. In addition, the present work did not include a systematic dose–response analysis or pharmacokinetic evaluation of the extract. Since the extract was not standardised to defined active compounds, potential batch-to-batch variation in chemical composition should also be taken into account when interpreting the reproducibility of the results. Finally, while our data support an association between the protective effect of the extract and regulation of the AMPK–mTOR–autophagy pathway, more rigorous mechanistic studies, such as rescue, knockdown, or inhibitor-based experiments, are still needed to verify pathway involvement at a causal level.

## Conclusions

This study investigated effects of *codonopsis* aqueous extract and its five major bioactive components on PEDV-induced autophagy in IPEC-J2 cells. The results demonstrated that *codonopsis* aqueous extract alleviated PEDV-induced cytopathic effects and ultrastructural damage, significantly suppressed PEDV N protein expression, and reduced *LC3* mRNA and protein expression. Both the aqueous extract and the individual compounds exerted antiviral effects by suppressing cellular autophagy through modulation of the AMPK/mTOR signaling pathway. The aqueous extract regulated this pathway through a balanced, multicomponent mode of action, whereas the individual components exhibited stronger target-specific inhibitory effects. Among them, apigenin showed the strongest antiviral and anti-autophagic activities, suggesting that it may represent a key antiviral constituent of *codonopsis*.

## Supplementary Information

Supplementary Material 1.

## Data Availability

Data will be made available on request.

## Abbreviations

PEDV: Porcine Epidemic Diarrhea Virus
TCM: Traditional Chinese medicine
DS, Dangshen: *Codonopsis pilosula*
SITO: β-Sitosterol
Que: Quercetin
HMF: 5-Hydroxymethylfurfural
APG: Apigenin
NA: Nicotinic acid
TGEV: Porcine enteric coronavirus
PoRV: Porcine rotavirus
PED: Porcine Epidemic Diarrhea
FBS: Fetal bovine serum
RIPA: Radioimmunoprecipitation assay
PVDF: Polyvinylidene fluoride
HRP: Horseradish peroxidase
CCK-8: Cell-Counting-Kit-8
DMEM: Dulbecco's Modified Eagle Medium
MOI: Multiplicity of infection
TCID_50_: Median tissue culture infective dose
PBS: Phosphate buffer saline
H&E: Hematoxylin and eosin
TEM: Transmission electron microscopy
DAPI: 4',6-Diamidino-2-phenylindole
ANOVA: Analysis of variance
LSD: Least significant difference
SD: Mean ± standard deviation
RER: Rough endoplasmic reticulum
