# Supplementary material for: Antiviral effects of Codonopsis pilosula extract and its bioactive components against porcine epidemic diarrhea virus in vitro via the AMPK/mTOR pathway
Source: BMC Vet Res. 2026 Apr 24;22:382. doi: 10.1186/s12917-026-05477-w (PMC13321427; doi:10.1186/s12917-026-05477-w)
Supplement: Supplementary file 1 — Supplementary Material 1. [file 12917_2026_5477_MOESM1_ESM.docx]

Antiviral Effects of *Codonopsis pilosula* Extract and Its Bioactive Components Against Porcine Epidemic Diarrhea Virus in vitro via the AMPK/mTOR Pathway

**Tao Ren ^a, b^** **^†^, Shiqin Pan ^a, †^, Xuqin Song ^a^, Liting Cao ^c^, Anchun Cheng ^a^, Yujie Zhan ^a^,**

**Jian Yang ^a, *^ and Deyuan Ou ^a,^[[1]](#footnote-1)^^**

(^a^ Institute of Veterinary Medicine and Immunology Drugs, Veterinary Department in College of Animal Science, State Key Laboratory of Green Pesticide, GuiZhou University, Guiyang 550025, China;

^b^ Qixingguan District Grassland Workstation, Bijie 551700, China;

^c^ Department of Traditional Chinese Veterinary Medicine, College of Veterinary Medicine, Southwest University, Rongchang, Chongqing, 402460, China)

**The full uncropped Gels and Blots image(s)**



DS-PEDV-12h（Figure 2-A）



DS-β-actin-12h（Figure 2-A）



DS-PEDV-24h（Figure 2-A）



 DS-β-actin-24h（Figure 2-A）



DS-PEDV-48h（Figure 2-A）



DS-β-actin-48h（Figure 2-A）

 DS-LC3-12h（Figure 2-D）



DS-β-actin-12h（Figure 2-D）


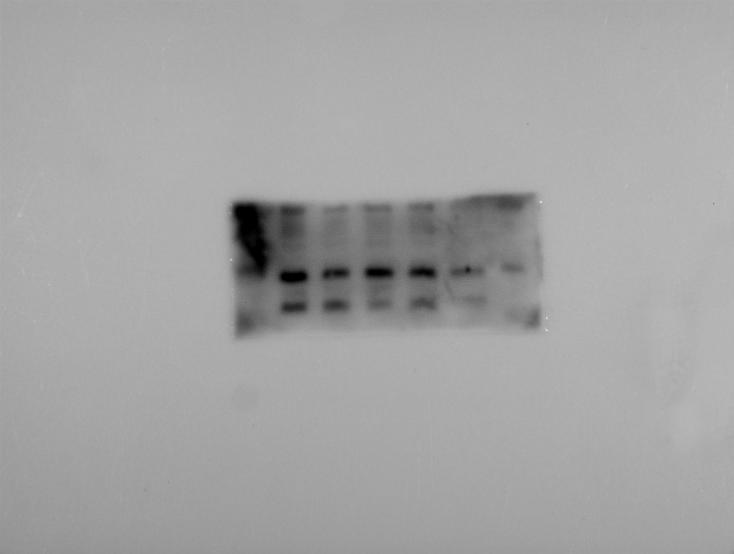
 DS-LC3-24h（Figure 2-D）


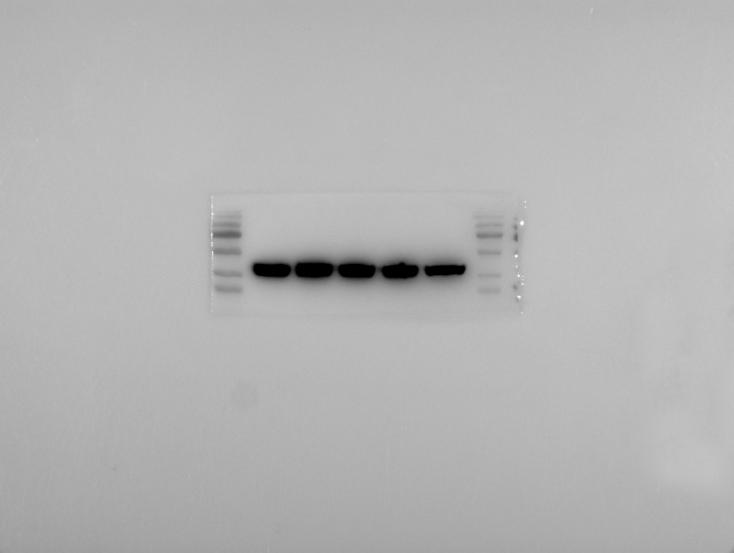
 DS-β-actin-24h（Figure 2-D）



 DS-LC3-48h（Figure 2-D）



 DS-β-actin-48h（Figure 2-D）


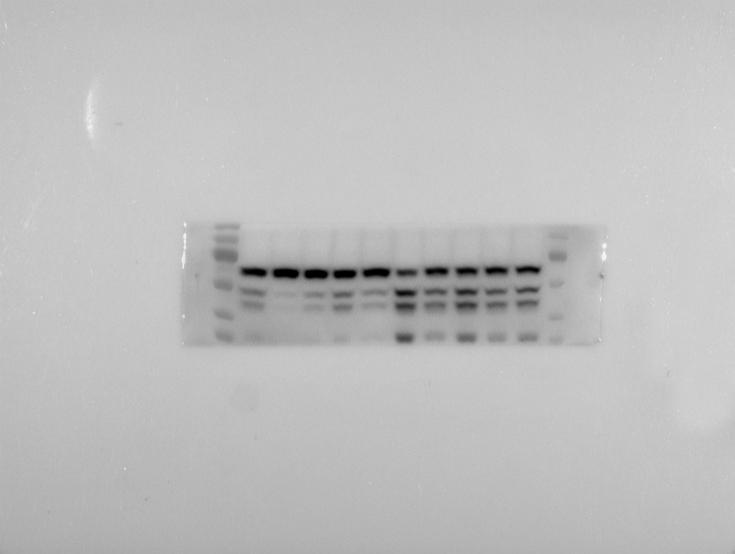
DS-AMPK-24h, 48h(Figure 3-D)


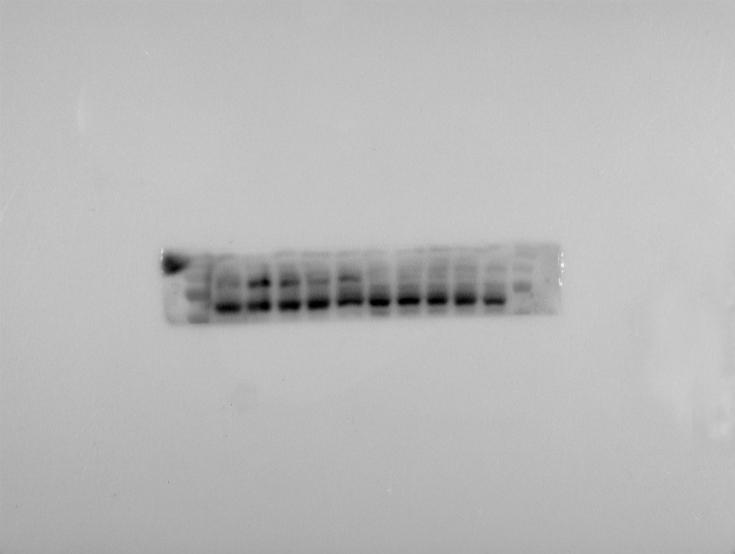
DS-p-AMPK-24h, 48h(Figure 3-D)


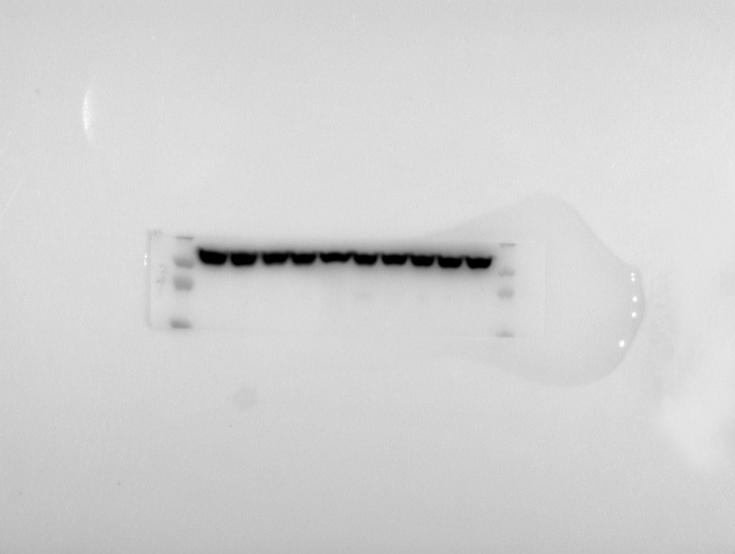
DS-β-actin -24h, 48h(Figure 3-D)


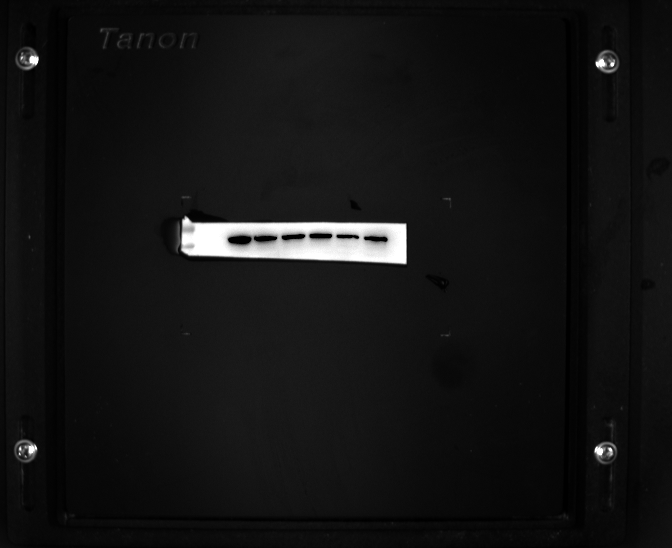
 Active components of Codonopsis -PEDV N -48h(Figure 5-B)


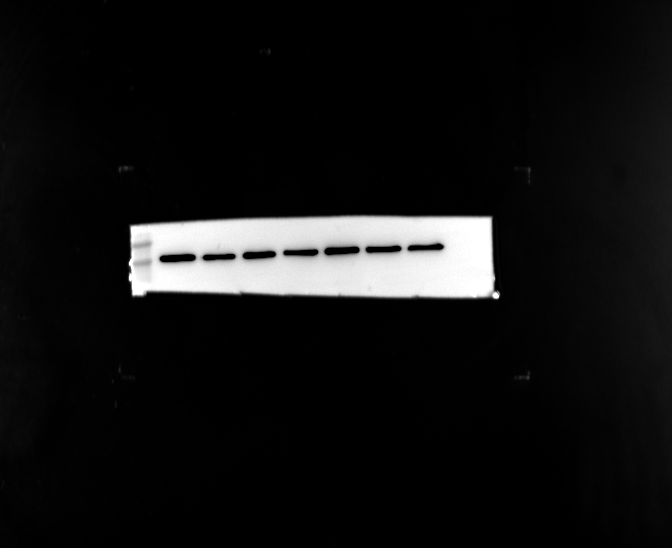
Active components of Codonopsis -GAPDH -48h(Figure 5-B) and (Figure 6-D)


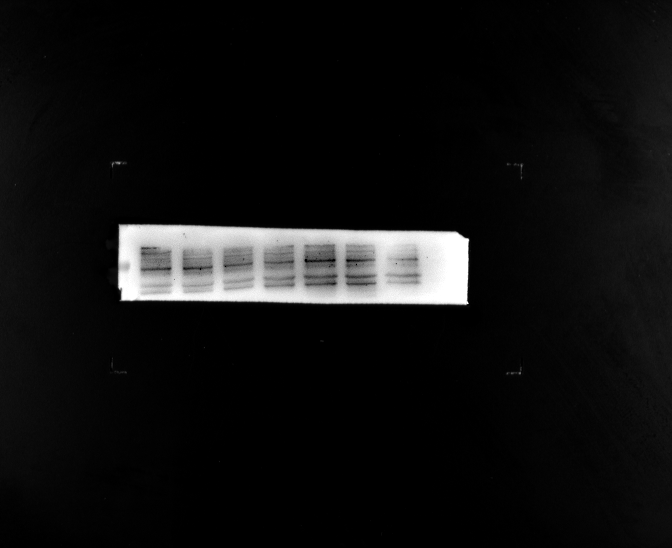
Active components of Codonopsis - AMPK -48h(Figure 6-D)


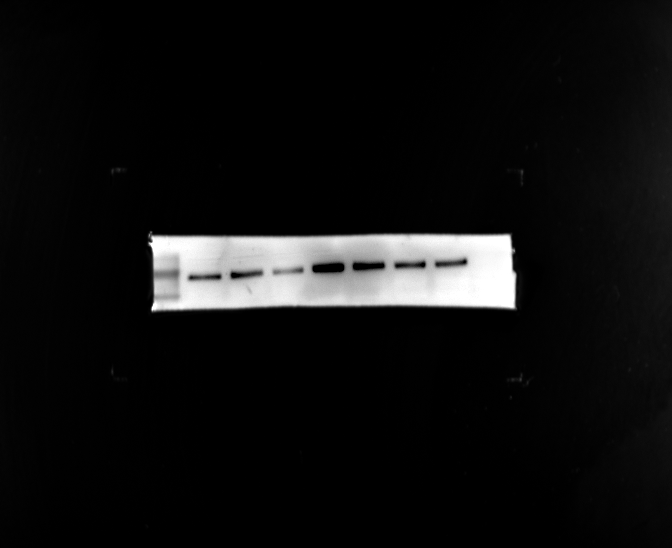
Active components of Codonopsis -p-AMPK -48h(Figure 6-D)


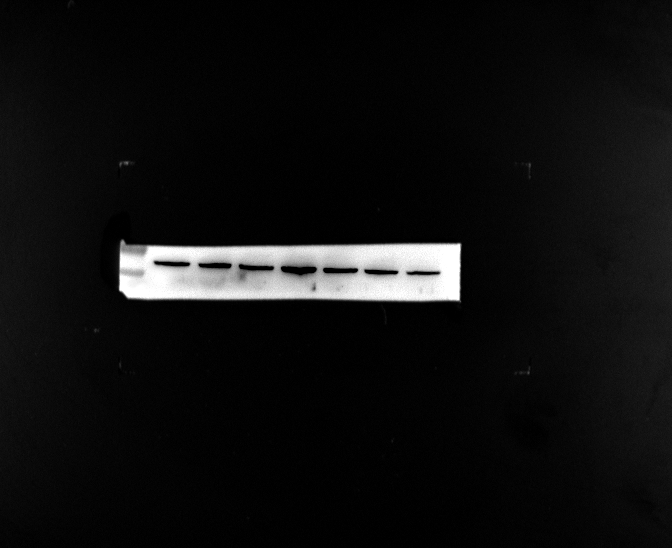
Active components of *Codonopsis* -ATG13 -48h(Figure 6-D)

1. Correspondence: cas.jyang@gzu.edu.cn; Co-correspondence: dyou@gzu.edu.cn; Tel.: 0851-88298005

   **^†^** The authors contributed equally to this work. [↑](#footnote-ref-1)
